# A reflection on polymer electrolytes for solid-state lithium metal batteries

**DOI:** 10.1038/s41467-023-40609-y

**Published:** 2023-08-12

**Authors:** Ziyu Song, Fangfang Chen, Maria Martinez-Ibañez, Wenfang Feng, Maria Forsyth, Zhibin Zhou, Michel Armand, Heng Zhang

**Affiliations:** 1 Key Laboratory of Material Chemistry for Energy Conversion and Storage (Ministry of Education), School of Chemistry and Chemical Engineering, Huazhong University of Science and Technology, Luoyu Road 1037, 430074 Wuhan, China; 2 Institute for Frontier Materials, Deakin University, Burwood, VIC 3125 Australia; 3 Centre for Cooperative Research on Alternative Energies (CIC energiGUNE), Basque Research and Technology Alliance (BRTA), 01510 Vitoria-Gasteiz, Spain

**Keywords:** Batteries, Materials for energy and catalysis, Energy supply and demand, Polymers, Electrochemistry

## Abstract

Before the debut of lithium-ion batteries (LIBs) in the commodity market, solid-state lithium metal batteries (SSLMBs) were considered promising high-energy electrochemical energy storage systems before being almost abandoned in the late 1980s because of safety concerns. However, after three decades of development, LIB technologies are now approaching their energy content and safety limits imposed by the rocking chair chemistry. These aspects are prompting the revival of research activities in SSLMB technologies at both academic and industrial levels. In this perspective article, we present a personal reflection on solid polymer electrolytes (SPEs), spanning from early development to their implementation in SSLMBs, highlighting key milestones. In particular, we discuss the SPEs’ characteristics taking into account the concept of coupled and decoupled SPEs proposed by C. Austen Angell in the early 1990s. Possible remedies to improve the physicochemical and electrochemical properties of SPEs are also examined. With this article, we also aim to highlight the missing blocks in building ideal SSLMBs and stimulate research towards innovative electrolyte materials for future rechargeable high-energy batteries.

## Abstract (web summary)

Polymer electrolytes are attractive candidates for rechargeable lithium metal batteries. Here, the authors give a personal reflection on the structural design of coupled and decoupled polymer electrolytes and possible routes to further enhance their performance in rechargeable batteries.

## Introduction

In his 1870 novel “Twenty Thousand Leagues Under The Sea” Jules Verne describes the submarine Nautilus as powered by an advanced battery system, and Captain Nemo mentions that “…the cells with sodium must be regarded as most energetic, and that their electromotive force is double that of the zinc cells.”^1^ The concept of building high-energy batteries proposed by Jules Verne was undoubtedly ahead of its time in the late 19th century but in line with the then fascination for the wonders generated by the use of electricity. “All with electricity” was a dream for humanity living in the early 1900s, but it would come into reality in the late 20th century with the invention of lithium-ion batteries (LIBs) based on the rocking chair concept (i.e., batteries built with two intercalation-based electrodes with different potentials for storing/delivering electrochemical energy)^2,3^. Presently, the global production of LIBs has reached a large scale of >500-gigawatt hour (GWh), acting as power sources for more than 6 million electric vehicles (EVs)^4^. The success of LIBs testifies to the early hypothesis of “all with electricity” and opens up new avenues for more sustainable development of energy-consuming anthropogenic activities.

The production capacity of LIBs has risen tenfold over the past decade^5^, and this demand is expected to continue growing rapidly over the next 10–30 years, driven mainly by the fast-growing EV sector^4^. The need for high-performance (e.g., energy density, safety, cost, etc.) rechargeable batteries is also pressing, particularly considering the stringent requirements brought by contemporary practical applications (e.g., EVs for road and flying, drones, advanced robotics, etc.), including inherent safety and specific energy (>500 Wh kg^−1^) and energy density (>1000 Wh L^−1^)^6^. Unfortunately, the nonaqueous liquid electrolytes used in today’s LIBs are unstable and highly flammable due to the presence of organic carbonate solvents (e.g., dimethyl carbonate, ethylene carbonate, etc.); additionally, the graphite negative electrodes with a relatively low specific capacity of 372 mAh g^−1^ are also limiting factors for improving further the specific energy of the state-of-the-art LIBs^6^. In this regard, solid-state lithium metal batteries (SSLMBs) coupling high-energy electrode materials (e.g., lithium metal (Li°), lithium alloys, nickel-rich LiNi_1−*x*−*y*_Co_*x*_Mn_*y*_O_2_ (1−*x* − *y* > 0.8), sulfur, etc.) with solid electrolytes are considered a viable approach to circumvent the specific energy density stumbling block of current LIB technology^7–9^.

Among all kinds of lithium-ion conductive solid-state electrolytes, solid polymer electrolytes (SPEs) have attracted significant attention owing to their physicochemical features (e.g., high flexibility, ease of thin-film processing)^10–12^. In particular, practical high-energy applications (>1 GWh) of SPE-based SSLMBs as power sources for EV and grid storage have been deployed by the Bolloré group since 2010^13^. This is a relevant industrial example of SPE technology capable of providing support for the development of high-performance SSLMBs.

Since the historical development of solid solution electrodes in LIB technologies is well reported in recent perspective and review articles^4,14–17^, here, we focus our attention on the evolution of SPE materials and their physical chemistries, with particular reference to their applications in rechargeable secondary batteries, aiming to bridge the gaps between the earlier-developed solid-state batteries and contemporary high-performance SPE-based SSLMBs. In particular, the timeline rationale of SPE-based SSLMBs evolution is discussed considering the concept of coupled and decoupled SPE systems introduced by C. Austen Angell in the early 1990s^18,19^.

### Birth of solid-state batteries

**Fig. 1:**
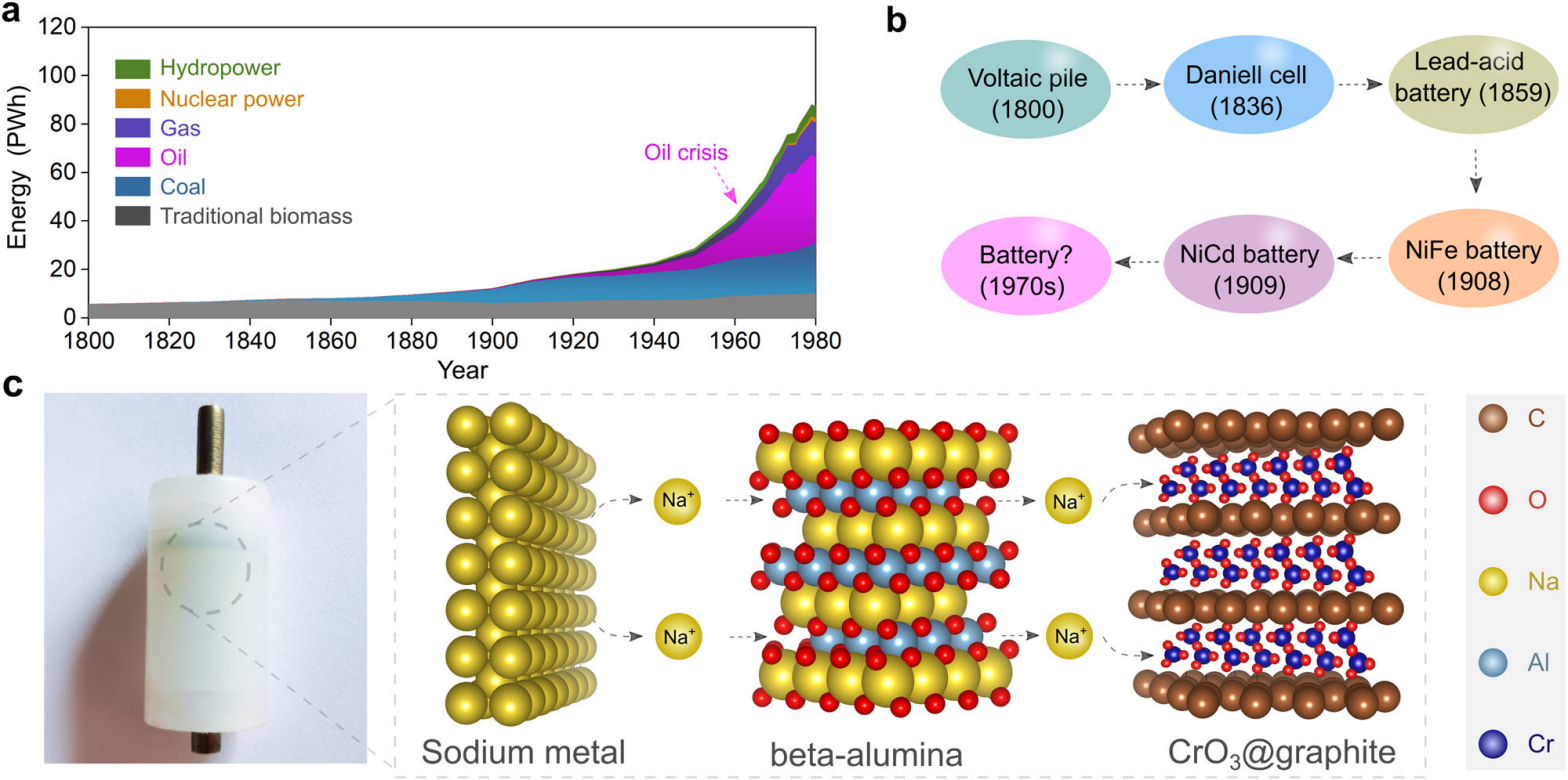
Towards the development of all-solid-state batteries. **a** Evolution of global energy consumption during the past two centuries. The dashed arrow notes a surge in oil consumption in the 1970s. **b** Brief outline of batteries developed before the 1970s, including the primary Voltaic pile, primary Daniell cell, secondary lead-acid battery, secondary nickel–iron alkaline battery, and secondary nickel-cadmium battery^27^. **c** Photographic picture of the first solid-state cell assembled in 1972, in which sodium metal and chromium oxide intercalated into graphite (CrO_3_@graphite) were utilized as negative and positive electrode active materials, respectively. The solid-state sodium-ion conductor β-alumina was adopted as the electrolyte for supporting the operation of solid-state cell at room temperature (25 °C) with moderate stacking pressure (ca. 10 MPa). The crystal structures of sodium metal, CrO_3_@graphite, and β-alumina are obtained from Materials Projects^120^ and re-constructed with VESTA software^121^.

Before the first industrial revolution in the early 19th century, global energy consumption relied heavily on traditional biomass (e.g., wood) and coal, as shown in Fig. 1a^20^. In the 1930s, significant advances in the petroleum industry enabled the shift from coal to petroleum-based high-energy sources^21^. However, petroleum resources are unevenly distributed worldwide, with nearly half located in the Middle East. So the increasing demand for petroleum-based energy from those countries having fewer reserves led to the oil crisis of the 1970s. This geopolitical situation prompted countries in the global north to explore alternative and disruptive technologies, seeking to transform the energy landscape and decrease dependence on fossil fuels, particularly through advancement in high-energy rechargeable batteries^22,23^.

Before the 1970s, the specific energy of rechargeable batteries remained lower than 50 Wh kg^−1^ at the pack level, significantly hindering their large-scale application in the automotive industry (Fig. 1b). The utilization of lithium or sodium metal (Na°) negative electrodes and other high-energy electrode materials was considered a straightforward and effective approach to improve the specific energy of rechargeable batteries. Before the early 1970s, several attempts had been made to recharge lithium metal-based high-energy batteries. However, the formation of electrically unstable lithium metal electrodeposition morphologies, such as dendrites formed during the cycling process, caused significant safety issues, including the risk of fire and explosion^24^.

It thus became clear that nonaqueous liquid electrolyte solutions with high volatility and flammability were incompatible with lithium metal negative electrodes. For using high-energy metal negative electrodes, a solid-state electrolyte was considered key to building high-energy rechargeable batteries operated in ambient temperature region (−40 to 40 °C)^25^. In 1972, the very first prototype of a sodium-based solid-state cell was assembled by M. Armand^25^ utilizing sodium metal as the negative electrode, β-alumina as the solid electrolyte, and a chromium oxide/graphite intercalation compound (CrO_3_@graphite) at the positive electrode. The physical image of the cell constructed 50 years ago is shown in Fig. 1c. Effectively, under room temperature (25 °C) and moderate stacking pressure (ca. 10 MPa), the all-solid-state Na°||CrO_3_@graphite cell would deliver a high theoretical specific energy of ca. 1000 Wh kg^−1^ at the material level (i.e., energy content calculated by the mass of CrO_3_@graphite), nearly three times higher than the nickel-cadmium batteries^26^.

### Discovery of solid polymer electrolytes

**Fig. 2:**
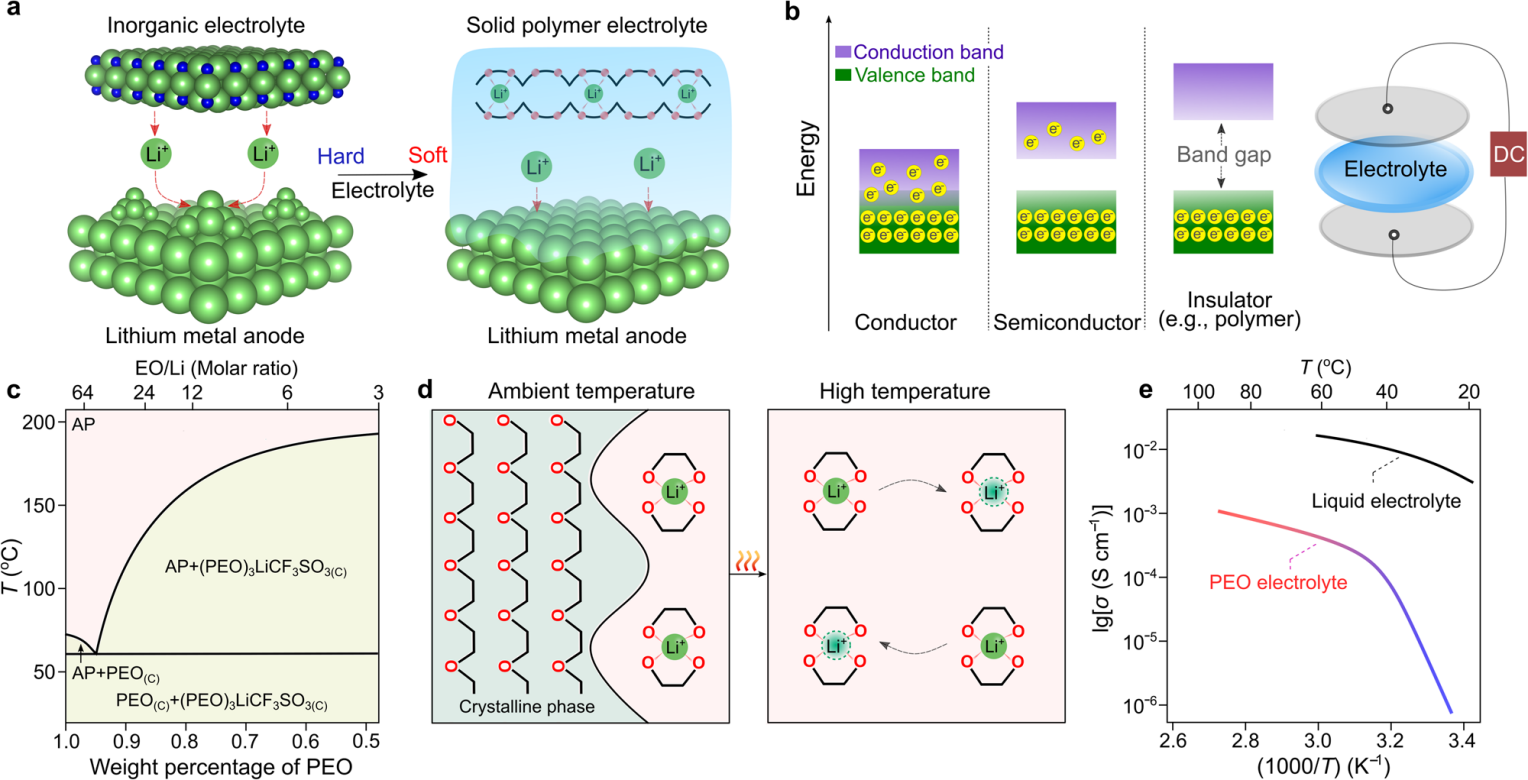
Comparison between solid polymer electrolytes (SPEs), inorganic solid electrolytes and nonaqueous liquid electrolyte solutions. **a** Critical role of soft contact between the electrode and a solid electrolyte. Lithium nitride (Li_3_N) and poly(ethylene oxide) (PEO)-based electrolytes are shown as typical examples for elucidating the distinctions between inorganic and organic materials. The crystal structures of Li_3_N are obtained from Materials Projects^120^ and re-constructed with VESTA software^121^. The light green, dark blue, and pink spheres refer to lithium, nitrogen, and oxygen atoms, respectively; and the black sticks represent the carbon–carbon linkage between two oxygen atoms in PEO. **b** Electron leakage versus ionic conduction in SPEs. Polymers were treated as insulating materials due to their large band gaps between valence and conduction bands (e.g., >4 eV for PEO^34^). The two discs (light gray) on the top and bottom of the SPE membrane represent the blocking electrodes. DC: direct current. **c** Phase diagram of lithium trifluoromethyl sulfonate (LiCF_3_SO_3_)/PEO. The values are taken from ref. ^38^. The light green and pink areas represent the amorphous phase (abbreviated as AP) region and two-phase region in the PEO-based electrolytes, respectively. PEO_(C)_ and (PEO)_3_LiCF_3_SO_3(C)_ denote the crystalline phase of PEO and the salt/polymer complex (i.e., (PEO)_3_LiCF_3_SO_3_), respectively. **d** Microscopic views of PEO-based SPEs at room (25 °C) and high (>60 °C) temperatures above the melting transition of PEO phases. **e** Effect of temperatur**e** on the ionic conductivity of PEO-based SPEs [(PEO)_20_LiCF_3_SO_3_] and conventional liquid electrolyte solutions (e.g., 1.0 mol kg^−1^ lithium hexafluorophosphate (LiPF_6_) per kilogram propylene carbonate). The ionic conductivity values are taken from refs. ^39,122^.

The first conference dedicated to solid-state materials was held in Belgirate (Italy) in 1972^27^, which greatly accelerated the development of solid-state batteries (e.g., silver metal|silver sulfide iodide|iodine cell)^28,29^. In addition to the already known sodium-ion conductors (e.g., β-alumina^30^), several kinds of inorganic electrolytes capable of transporting lithium ions were discovered before the mid-1970s, including lithium iodide (LiI)^31^ and lithium nitride (Li_3_N)^32^. However, the high mechanical stiffness of inorganic electrolytes (e.g., Young’s modulus of 150 GPa for Li_3_N^32^) results in inadequate physical contact between the electrodes and the inorganic electrolyte, as illustrated in Fig. 2a. A high Young’s modulus also makes inorganic electrolytes unable to accommodate the induced mechanical stresses resulting from volume changes during cycling, resulting in cracks/small gaps formation at the electrode|electrolyte interface and interphases, the evolution of parasitic side reactions and formation of unfavorable metallic electrodepositions (e.g., dendrites)^33^.

To overcome the contact issues between two rigid materials, designing soft solid electrolytes is an intuitive solution. In the late 1960s, it was established that polymeric materials are good electronic insulators (i.e., materials with the capability of blocking the transport of electrons) with large band gaps (>4 eV^34^) for electron jumping between conduction and valence bands (Fig. 2b). Yet, it was not clear whether the transportation of ionic species would be possible at that time. In 1966, Lundberg et al.^35^ investigated the mixture of metal salts (e.g., potassium iodide) and poly(ethylene oxide) (PEO). They concluded that metal salts interact with PEO and reduce crystallinity. In 1971, M. Armand carried out several ionic conductivity tests with lithium bromide (LiBr)/PEO. From the analysis of the results, he concluded that because of the very high resistance (>1 MΩ) measured at room temperature (ca. 20–30 °C), the utilization of LiBr/PEO for battery applications was not recommended. Two years later, Fenton et al.^36^ discovered that the mixtures of PEO and low-lattice-energy metal salts (e.g., sodium iodide (NaI), sodium thiocyanate (NaSCN), potassium thiocyanate (KSCN), etc.) become ionically conductive upon warming up the samples (e.g., ionic conductivities for the (PEO)_4_KSCN complex: 10^−7^ (40 °C) vs. 10^−2^ S cm^−1^ (170 °C)). This key finding rapidly caught the attention of Armand, and he suggested the utilization of these polymeric ionic conductors as solid electrolytes for building solid-state batteries^37^. These pioneering research works ushered a new direction for developing soft solid electrolytes and circumventing the surface contact issue in solid-state batteries with inorganic solid electrolytes.

However, it was still unclear why the ionic conductivity of PEO-based SPEs was sensitive to temperature. To shed some light on this aspect, Vallée et al. and Robitaille et al.^38,39^ systematically studied the phase diagrams of a series of lithium salt/PEO binary mixtures and revealed that PEO forms crystalline complexes with various kinds of lithium salts (e.g., (PEO)_3_lithium trifluoromethanesulfonate (LiCF_3_SO_3_), (PEO)_6_lithium perchlorate (LiClO_4_)) and eutectic mixtures with low melting transitions from 40 to 60 °C depending on the type of salt anions (e.g., 55 °C for (PEO)_32_LiCF_3_SO_3_), as shown in Fig. 2c.

Using solid-state nuclear magnetic resonance spectroscopy, Berthier et al.^40^ demonstrated that the amorphous phases in the PEO-based SPEs are primarily responsible for the transport of ionic species within the SPE. Therefore, the presence of crystalline phases at room temperature (e.g., 20–30 °C, Fig. 2d) was indicated as the main reason for the low ionic conductivities of PEO-based SPEs. Figure 2e shows the comparison of ionic conductivities of LiCF_3_SO_3_/PEO and lithium hexafluorophosphate (LiPF_6_)/propylene carbonate, which are representative examples of SPEs, and conventional nonaqueous liquid electrolyte solutions, respectively. In particular, the LiCF_3_SO_3_/PEO electrolyte shows two different regions below and above the melting transition of the crystalline phases in the Arrhenius plot, which highlights the critical role of the testing temperature on the ionic conductivity of the PEO-based SPEs.

### Coupled SPEs: from classic PEO to other emerging systems

**Fig. 3:**
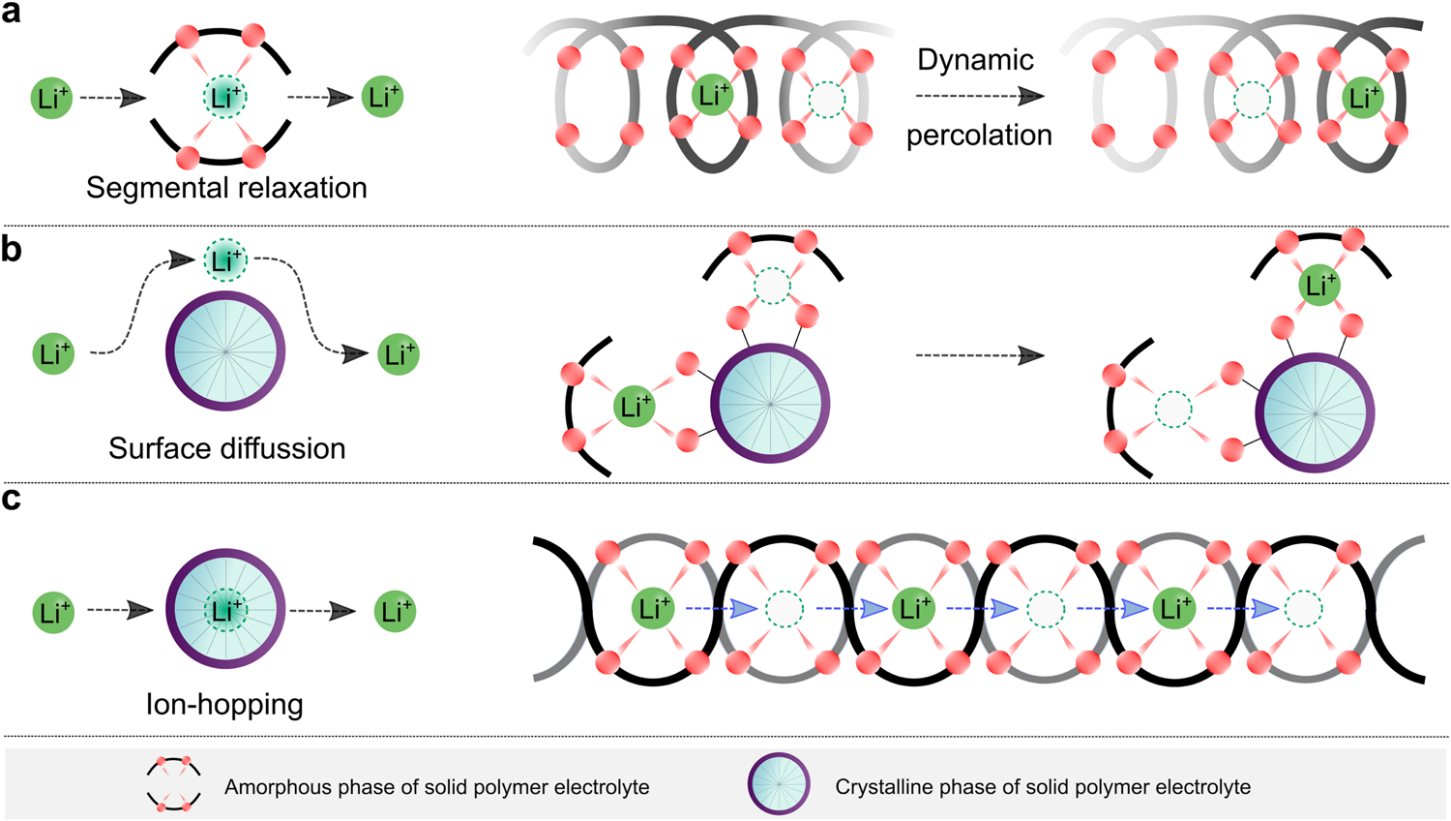
Transport of ionic species in different coupled solid polymer electrolytes (SPEs). **a** Graphical representation of the microscopic transport of lithium ions in fully amorphous phases, in which lithium-ion migration is tightly correlated with the segmental dynamic of polymer backbones. **b** Graphical representation of the microscopic transport of lithium ions in the mixture of amorphous and crystalline phases, where the surface functional groups of crystalline phases favor the transport of ionic species. **c** Graphical representation of the microscopic transport of lithium ions in crystalline phases, in which the cationic species migrate via the ion-hopping mechanism.

For early-developed PEO-based SPEs, the ion transport at the microscopic scale is illustrated in Fig. 3. Generally, in amorphous phases, the long-range transport of ionic species, particularly lithium ions, is mainly realized via a segmental motion of the polymer backbone (Fig. 3a), following the percolation model proposed by M. A. Ratner and co-workers^41^. In the mixture of amorphous and crystalline phases, the crystalline surface can assist ion transport through surface coordination (Fig. 3b)^42^. In contrast, the inner cores of crystalline phases do not allow the lithium-ion transport due to the immobilized polymer segments, like in the helical conformation of PEO in the crystalline structure of (PEO)_3_LiCF_3_SO_3_^43^. It is accepted that most crystalline complexes are poor ionic conductors, and the presence of these crystalline spherulites (i.e., typical morphology of crystalline PEO) in PEO-based SPEs causes a significant drop in ionic conductivities by discontinuing the conduction pathways in the amorphous phases^44^. For a well-defined crystalline complex (e.g., (PEO)_6_lithium hexafluoroarsenate), ion transport becomes possible via the hopping of lithium ions to adjacent sites (Fig. 3c)^45^. It has to be stressed that the molecular weight of PEO and the type of lithium salts are crucial to guarantee the rapid transport of lithium ions since the ion-hopping pathways are strongly associated with the accessible defects within these crystalline polymers.

**Fig. 4:**
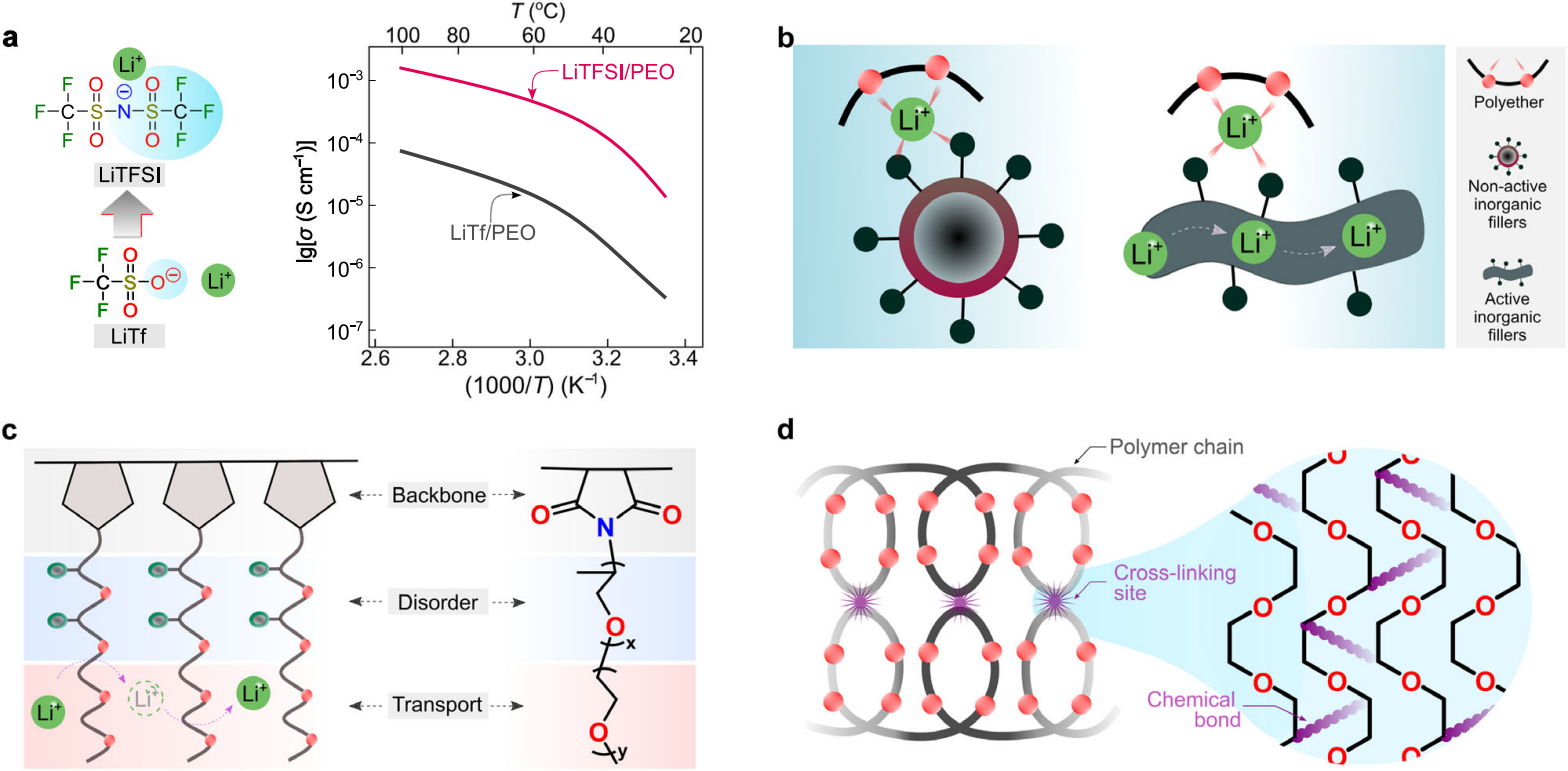
Key steps in the development of coupled solid polymer electrolytes (SPEs). **a** Chemical formula of lithium bis(trifluoromethanesulfonyl)imide (LiTFSI) and lithium trifluoromethanesulfonate (LiTf) and their ionic conductive properties in poly(ethylene oxide) (PEO)-based solid electrolytes at various temperatures. The values are taken from ref. ^38^. **b** Graphical representation of the inclusion of inorganic fillers in polymer electrolytes. **c** Schematic representation of amorphous Jeffamine-based polymer electrolytes. **d** Graphical summary of the in situ ultraviolet photo-irradiated cross-linking strategy for the PEO-based electrolytes.

Because in the two former cases (Fig. 3a, b) the lithium-ion transport is associated with the segmental motion of PEO, these systems are labeled as coupled SPEs. In these cases, the degree of lithium salt dissociation and the structural flexibility (i.e., ease in conformational change) of anions determine the ionic conductivities of SPEs. Before the 1980s, the salts employed for SPEs contained mainly inorganic anions (e.g., SCN^−^(see ref. ^36^) and ClO_4_^−^(see ref. ^46^)), which either bind too strongly to lithium ions or have poor structural flexibility. The available organic anions at the time, e.g., CF_3_SO_3_^−^, also bind tightly to lithium cations, as demonstrated by vibrational spectroscopic studies of PEO and LiCF_3_SO_3_ mixtures^47^. Thanks to the development of sulfur-nitrogen chemistry^48^, in 1972, Meussdorffer et al.^49^ reported the preparation of the bis(trifluoromethanesulfonyl)imide (TFSI^−^) anion with a highly delocalized negative charge and inherent structural flexibility. This molecule was then brought into the battery research field by Armand et al.^50^ in the early 1980s. As seen in Fig. 4a, replacing CF_3_SO_3_^−^ with TFSI^−^ in the salt anion leads to an order of magnitude increase in total ionic conductivity (i.e., the sum of cationic and anionic conductivities), reaching 10^−3^ S cm^−1^ above the melting transitions of PEO phases (e.g., ca. 2 × 10^−3^ at 100 °C)^38^. This ionic conductivity meets the minimum requirements (i.e., >10^−3^ S cm^−1^) for operating SPE-based SSLMBs at elevated temperatures (≥80 °C)^51,52^. In the last decade, the development of molecules with delocalized negative charges has further progressed^53,54^. For example, Ma et al.^54^ proposed a delocalized polyanion, i.e., poly[(4-styrenesulfonyl)(trifluoromethyl(*S*-trifluoromethylsulfonylimino) sulfonyl)imide] (PSsTFSI^−^), that demonstrates improved lithium-ion conductivity of SPEs for unipolar conduction (i.e., only positive charges are mobile) due only to lithium cation (e.g., at 80 °C, ca. 10^−4^ S cm^−1^ for LiPSsTFSI-based electrolyte and ca. 10^−5^ S cm^−1^ for lithium poly[(4-styrenesulfonyl)(trifluoromethanesulfonyl)imide] (LiPSTFSI)-based electrolyte^54^). The polyanion PSsTFSI^−^ could be obtained through the replacement of an oxygen atom in a TFSI-like moiety (i.e., CF_3_SO_2_N^(−)^SO_2_—) with strong electron-withdrawing trifluoromethanesulfonylimino ( = NSO_2_CF_3_) group; thus, the negative charges are further delocalized via five oxygens and two nitrogen atoms. These research works demonstrate an effective strategy for improving the ionic conductivity in coupled SPEs by weakening the interaction between salt anion and lithium ions.

Alternatively, to further increase the conductivity of SPEs, Croce et al.^55^ suggested the utilization of nano-sized inorganic fillers as active sites for suppressing the crystallization of ethylene oxide (EO) chains and promoting the lithium-ion transport via surface mechanism (Fig. 4b). Inorganic fillers can be electrochemically non-active or active (e.g., garnet and lithium phosphorus oxynitride)^56^, in which the bulk phases of inorganic fillers could provide additional transport channels besides the polymer phases (Fig. 4b). In addition, to facilitate rapid lithium-ion transport, the morphology of inorganic fillers was expanded from nanoparticles (i.e., less than 100 nm for all three Cartesian dimensions of the nanoparticle) to nano-wires (i.e., less than 100 nm for the diameter of the nanowire)^57,58^. Functionalization of nanofiller was also suggested to improve the compatibility of organic and inorganic phases^59^. It has to be noted that experimental evidence for the transport of lithium ions between polymer and inorganic phases is not widely reported, and this mechanism is not fully understood. However, it is reported that the transport along inorganic bulk phases is likely to only occur in inorganic-rich (>50 vol%) SPEs or when particular morphologies (e.g., nanowire) of inorganic phases are used^56^.

Various modification strategies, including polymer blends and co-polymerization^12^, have been proposed in the last two decades to suppress the crystallization of PEO matrices. For example, the co-polymerization of EO-containing monomers with styrene-based monomers has been investigated by various researchers^60–62^. It has been concluded that incorporating styrene-based moieties effectively suppresses the crystallization of EO units and improves the mechanical properties of the as-formed membranes^61,62^. Yet, sophisticated synthetic procedures are needed to tailor the chemical structures of the polymer matrices, which hinders the practical deployment of such strategies.

Jeffamine® compounds are commercial amine-terminated polyether oligomers produced by Huntsman Corporation^63^, widely used as foam stabilizers and corrosion inhibitors in petroleum industries. In 1992, Benrabah et al.^64^ suggested the utilization of the Jeffamine moiety as a charge carrier region for SPEs. Systematic investigations were also carried out to optimize the Jeffamine moiety’s chemical structures. It was found that the Jeffamine-based comb-like polymers (i.e., polymers comprising a linear backbone grafted with multiple side chains^65^) remain amorphous at room temperature (e.g., 20–30 °C) due to the presence of structurally disordered propylene oxide (PO) units (Fig. 4c), allowing an order of magnitude increase in ionic conductivities at room temperatures (e.g., 20–30 °C) for the corresponding SPEs compared to the PEO-based solid electrolytes^66^. Replacing LiTFSI with lithium bis(fluorosulfonyl)imide (LiFSI) in Jeffamine-based electrolytes leads to improved chemical and electrochemical stabilities of the lithium metal negative electrode, enabling the operation of SPE-based SSLMBs close to room temperatures (e.g., 20–30 °C)^67^.

To enhance the mechanical strength of PEO-based SPEs, Kim et al.^68^ reported in situ ultraviolet photo-irradiated cross-linking of PEO-based electrolytes in the absence of volatile solvents (Fig. 4d). This SPE preparation strategy could also be considered for possible implementation during hot-pressing processes (i.e., pressing electrolyte materials between two hot plates) which are typical procedures for making thin SPE films^69^. The preparation of cross-linked polymers through other synthetic methods has also been reported, as well as crystallinity suppression, ionic conductivity increment below the PEO melting temperature, and improvement of the elastic properties of SPEs^33,70^. In the past decades, the structural optimizations of lithium salts and polymer matrices have improved the transport, mechanical, and interfacial properties of the coupled SPEs, endowing the SPE-based SSLMBs with stable and highly reversible battery cycling performances^12^.

### Decoupled SPEs: old fashion and emerging systems

**Fig. 5:**
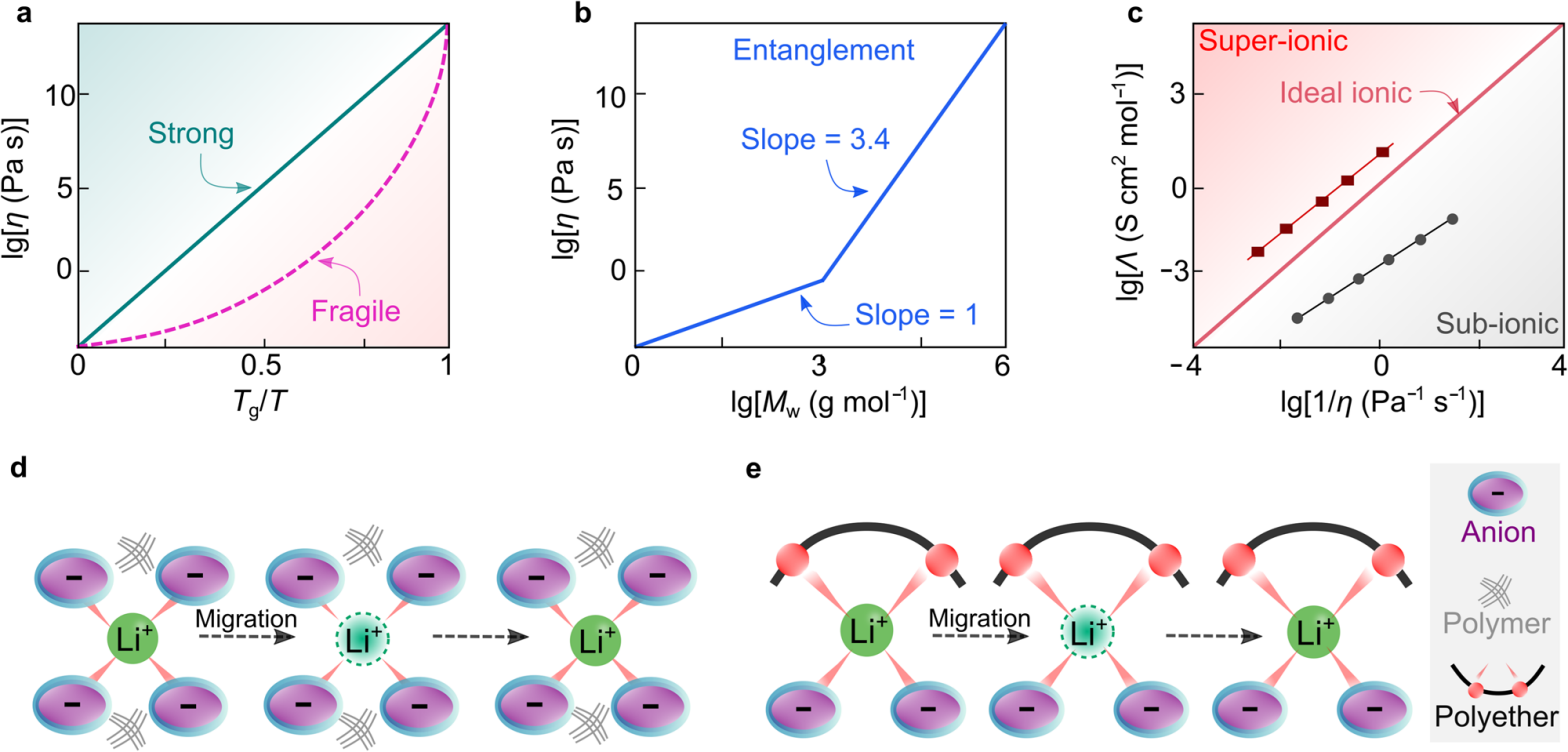
Decoupling the transport of ions from polymer dynamics. **a** Angell plot for strong and fragile solid materials. **b** Entanglement mechanism of polymeric materials. Adapted from ref. ^71^ with permission. Copyright 1998 Society of Chemical Industry. **c** Walden–Angell plot for ionic materials. The values are taken from ref. ^18^. **d**, **e** Schematic representation of the microscopic ionic transport in true (**d**) and pseudo (**e**) decoupled solid polymer electrolytes. For true decoupled systems, polymers (black lines) empower the as-formed electrolytes with certain mechanical strength without participating in the transport of lithium ions.

The main shortcomings of a strongly coupled SPE system are the low ionic conductivity in response to high *T*_g_ and the low lithium-ion transference number (*t*_Li_^+^). Typically, the value of *t*_Li_^+^ represents the portion of total currents carried solely by lithium ions. For coupled SPEs, the lithium cations are highly coordinated by electron-donating groups (e.g., ether oxygens) and become less mobile than the corresponding free anionic species (i.e., uncoordinated anions), resulting in relatively low metal-ion transference number (i.e., *t*_M_^+^ <0.5). As discussed above, a straightforward approach to increasing the ionic conductivity of coupled SPE is mainly through decreasing glass transition temperatures (*T*_g_) via structural manipulation. Another approach proposed in refs. ^18,71^ is to look for more fragile polymers, which can be assessed by C. Austen Angell’s plot (Fig. 5a). Here, the term fragile signifies a deviation from Arrhenius behavior. Glass-formers with a high fragility are called fragile, that is, experiencing a more rapid increase in viscosity (i.e., faster cooling process) as they approach the glass transition temperature *T*_g_ (Fig. 5a)^18^. At the same temperature above *T*_g_, the higher fragility of polymer, the larger deviation from Arrhenius behavior^18,71^. This eventually leads to higher ionic conductivities for the SPE comprising “fragile” polymer vs. conventional polyethers. For example, poly(vinyl chloride) (PVC) tends to be more fragile than poly(isobutylene), and in principle, the use of PVC could increase ionic conductivity. Since the glass transition temperature of polymer also strongly affects the ionic conductivity in coupled SPEs, finding an SPE capable of simultaneously satisfying the low *T*_g_ and high-fragility requirements is challenging, which limits a broader application of this fragility concept in SPEs.

Nevertheless, given the solid nature of SPEs, the increment of the polymer segmental motion alone cannot enhance metal-ion diffusion enough unless the metal-ion motions are decoupled from the polymer dynamics (e.g., segmental diffusion, chain relaxation, etc). Some attempts to enhance the decoupled metal-ion motion included modifying the polymer chemistry in the backbone by incorporating functional groups that weakly coordinate with metal ions, such as poly(carbonates)^72^ and poly(tetrahydrofuran)^73^. However, this strategy has so far failed to significantly improve ion decoupling and ionic conductivities. Indeed, the ionic conductivity remained low due to the high rigidity of polymer backbones^74^.

In the 1990s, Angell et al.^75^ proposed a novel approach for achieving decoupled superionic systems (i.e., the ion transport which is decoupled from the apparent viscosity of system) via a so-called polymer-in-salt (PIS)-type SPEs. In this work, a high content (90 wt%) of a superionic four-salt melt (i.e., LiI/lithium acetate (LiOAc)/lithium chlorate (LiClO_3_)/LiClO_4_) was mixed with a small amount (10 wt%) of high-molecular-weight (4 × 10^3 ^g mol^−1^) poly(propylene oxide), achieving a room temperature (e.g., 20–30 °C) ionic conductivity of about 10^−4^ S cm^−1^. The salts provide ionic conductivity and the role of polymer here is to provide mechanical strength. According to ref. ^76^, the macroscopic viscosity (i.e., apparent viscosity which is experimentally measurable) of polymers increases significantly when the numbers of repeat units exceed a threshold value of 200, which is known as the entanglement effect (Fig. 5b). Such a distinctive feature allows the solidification of this superionic liquids system upon the addition of small amounts (<5 wt%) of high-molecular-weight polymers.

The concept of decoupling in such a system is formulated on a hypothesis that when the salt concentration keeps increasing to a threshold, *T*_g_ finally will reach a maximum, then further increasing salt concentration will enhance the ion movements and should simultaneously increase the decoupling of the small metal ions from the polymer backbone. Later in the early 2000s, research provided further insight into the PIS electrolytes. It was shown that excess salt in SPEs leads to the formation of salt aggregates in which metal ion can diffuse through anions. Those aggregates become more interconnected at high salt concentrations (>50 wt% of salt in SPEs)^17^, promoting the metal ions to diffuse through this second conduction path. This demonstrates the ability of PIS-type SPEs to decouple metal-ion motion from polymer dynamics^77^. Several criteria, such as polymer *T*_g_, salt type, polymer/salt solubility, electrochemical stability, and ionic conductivity^78^, were also discussed in C. Austen Angell’s early works to understand the physicochemical properties of PIS electrolytes. Among these, the concept of the ionicity of lithium salt is of utter importance. Specifically, ionicity is a measure of the degree of ion dissociation, commonly referring to the effective fraction of ionic species being able to participate in ionic conduction^18^. Figure 5c displays the Walden-Angell plot for the dependence of equivalent conductivities on the viscosities of electrolytes. With 1.0 M potassium chloride/H_2_O solution as a reference electrolyte, the regime above the diagonal line refers to the electrolyte materials with super-ionic characters. For PIS-type SPE systems, the lithium salt should possess sufficient ionicity to ensure the high conductivity, i.e., be located in the super-ionic regime in Fig. 5c.

However, the superionic salt solution C. Austen Angell proposed is not applicable to lithium metal batteries due to the poor chemical and electrochemical stabilities of the as-prepared PIS-type SPEs. Starting from the late 1990s, several other PIS-type SPE systems having different polymer structures and salts were proposed and investigated, including poly(acrylonitrile) (PAN)-in-salt^79^, which incorporated a LiCF_3_SO_3_ salt with the PAN polymer. Unfortunately, this PIS electrolyte showed inadequate ionic conductivities, e.g., 2 × 10^−6^ S cm^−1^ at 50 °C and 10^−5^ S cm^−1^ at 75 °C^79^, which were increased to 10^−4^ S cm^−1^ at 30 °C by Wu et al.^80^ in 2016 used graphene oxide (GO) as a nanofiller. The authors claim that the GO nanosheets provide a fast three-dimensional ion transport network. In addition, recent research works reported various benefits of using PIS-type electrolytes for lithium metal batteries, including but not limited to stable interphase formation, improved cycling performance^81^, enhanced oxidation stability^82^, and increased compatibility with high-voltage positive electrodes^83^.

However, the proposed PIS electrolytes are still far from achieving true decoupling. Indeed, in a true decoupled system, metal ions should move independently of the polymer (Fig. 5d)^18^. In practical non-ideal systems (*aka*. pseudo-decoupled systems), true decoupling cannot be obtained because of the chemical interactions constantly occurring between ions and the repeat units (Fig. 5e). Therefore, in a decoupled superionic system, the influence of polymers on the motion of metal ions should be minimized, and the ionicity of salt should be maximized. Experimentally, one can measure decoupled motion by comparing time scales for polymer structural relaxation (i.e., cooperative reorientation of polymer segments^78^) with ionic conductivity relaxation using *T*_g_-scaled Arrhenius plot^84^. So far, the search for superionic low melting-point salts for alkali metal ions is still ongoing and represents a great challenge for the electrolyte research community^85^. Nevertheless, the strategy of using salt mixtures to maintain their liquid state at room temperatures (e.g., 20–30 °C) to achieve high ionic conductivities was successful^85^. Clearly, C. Austen Angell’s research opened a new avenue for designing solid and liquid electrolyte materials^86,87^.

**Fig. 6:**
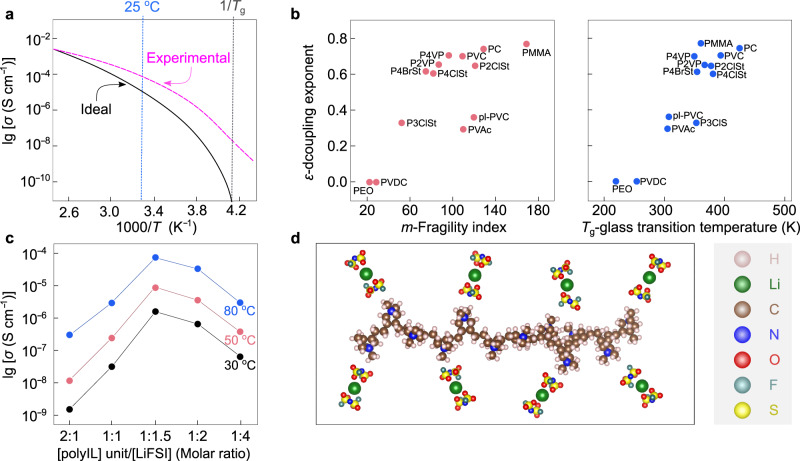
Properties of some representative decoupled polymer electrolytes. **a** Comparison between ideal and experimental decoupled solid polymer electrolytes (SPEs) in a typical Arrhenius plot for ionic conductivities. Adapted from ref. ^71^ with permission. Copyright 1998 Society of Chemical Industry. **b** Dependence of decoupling exponent (*ε*) on fragility index (*m*) and glass transition temperature (*T*_g_) for various kinds of polymers. pl-PVC plasticized poly(vinyl chloride), PC poly(carbonate), PEO poly(ethylene oxide), PMMA poly(methyl methacrylate), PVAc poly(vinyl acetate), PVC poly(vinyl chloride), PVDC poly(vinylidene chloride), P2ClSt poly(2-chlorostyrene), P2VP poly(2-vinylpyridine), P3ClSt poly(3-chlorostyrene), P4BrSt poly(4-bromostyrene), P4ClSt poly(4-chlorostyrene), P4VP poly(4-vinylpyridine). Adapted from ref. ^90^ with permission. Copyright 2011 American Chemical Society. **c** Ionic conductivities of poly(diallyldimethylammonium) (PDADMA)-based electrolytes vs. concentration of lithium salts at 30 °C (black line), 50 °C (red line), and 80 °C (blue line). Reproduced from ref. ^95^ with permission. Copyright 2019 Elsevier. **d** Interactions between lithium cations and anions in the P(DADMA^+^)-based electrolytes. The chemical structures are visualized with VESTA software^122^.

In the field of electrolytes, the equivalent conductivity of a given system is generally found to be inversely proportional to its viscosity, as defined by the Walden rule (Eq. (1))^88^

1 $$\varLambda \eta={constant}$$

where *Λ* is equivalent conductivity, and *η* is viscosity. Effectively, an ideal decoupled electrolyte follows the Walden rule and shows a certain degree of derivation from the Arrhenius behavior in the ionic conductivity–temperature, provides significantly increased ionic conductivity as temperature elevates slightly above glass transition temperature (Fig. 6a, black line). However, due to the entanglement effect (i.e., transient cross-links between polymer chains), the Walden rule becomes unapplicable for decoupled SPEs, and the ionic conductivities remain at much higher values than expected (i.e., viscosity does not dominate the transport of the ions; Fig. 6a, red line). Therefore, the breakdown of the Walden rule (i.e., diminishing the impact of viscosity on ion transport) effectively favors the increase of ionic conductivity for SPEs.

Agapov et al.^89^ systematically investigated the relations between the decoupling exponent (*ε*), fragility index (*m*), and *T*_g_ (see Fig. 6b). The decoupling exponent and fragility index are mathematically expressed below^90,91^:

2 $$m\propto \left.\frac{{{{{{\rm{d}}}}}}[\log \uptau]}{{{{\mbox{d}}}}\frac{{{{{{\mathrm{Tg}}}}}}}{{{\mbox{T}}}}}\right | _{{{{{{\rm{T}}}}}}={{{{{\mathrm{Tg}}}}}}}$$

3 $$\frac{{({\sigma }_{{{\mbox{o}}}}\times T)}^{-1}}{\tau }\propto {\tau }^{-\upvarepsilon}$$

Where *ε* is the decoupling exponent, *τ* is the characteristic relaxation time of the polymer segment, *T*_g_ is the glass transition temperature, *σ*_o_ is the ionic conductivity at temperatures close to *T*_g_, and *T* is the temperature^89^. It has been demonstrated that ionic species might migrate in high-fragility polymers (fragility index *m* > 40^89^) via the loose structures (i.e., rigid polymer chains with low packing density), despite their low segmental relaxation rate; segmental motion is necessary for the less-fragile polymers with dense structures (i.e., compact packing of flexible polymer chains), including PEO and other polyethers.

In 2017 and 2019, Angell wrote two review articles to discuss the research directions to achieve decoupling ion motion^18,84^, namely, the side-chain solvation decoupling and the anion-trapping strategies. The concept of side-chain solvation decoupling is to allocate the cation-solvating groups on a pendant side chain of polymer matrices to decouple the transport of cations from the segmental relaxation of the polymer backbone. Unfortunately, the practical implementation of this strategy is challenging due to the absence of the secondary relaxation effect (e.g., the motion of side chains) before reaching sufficient relaxation time (e.g., 10^−10^ s) required by ion transport for battery use^84^. In comparison, anion-trapping strategies have shown some success in increasing *t*_Li_^+^ values in polymer electrolytes either by developing polymers that are more prone to solvate anions than lithium ions (such as including under-coordinated boron centers in polymer chains^92^) or designing new anion chemistries to increase its interaction with polymers and slow down anion motion^93^. A more direct method to trap anions can be using a cationic polymer electrolyte^94^. However, the recent progress in developing cationic polymer electrolytes is not only limited to providing anion-trapping functions.

For example, Wang et al.^94^ reported a type of PIS-type SPE based on cationic poly(ionic liquid) (PolyIL, Fig. 6c, d), in which ionic liquid cations are chemically bound to a polymer backbone, showing promising physicochemical and electrochemical results. This PolyIL, more precisely poly(diallyldimethylammonium) bis(fluorosulfonyl)imide (P(DADMA^+^)FSI^−^), presented a decreased *T*_g_ at elevated LiFSI salt concentrations from a low to a high concentration range ([Li^+^]/[polycation] increases from 0.5 to 4). The highest conductivity was obtained at a high salt concentration of [Li^+^]/[polycation] = 1.5 within the temperature range from 30 °C to 80 °C, which in fact, forms a PolyIL-in-salt (PolyIL-IS) electrolyte. Later, through molecular simulations, Chen et al.^95^ predicted the fast transport of sodium- and potassium- ions in the same PolyIL system. They also experimentally verified the sodium-ion conduction behavior for the PolyIL-IS [Na^+^]/[polycation] = 2 system. The PolyIL-IS system demonstrated an ionic conductivity of 1 × 10^−3^ S cm^−1^ at 80 °C without adding any plasticizers (i.e., compounds for improving polymer dynamics), and a high decoupling index with log (*R*_*τ*_) close to 6.3, which is clear evidence to suggest the decoupled ion motion in this PolyIL-IS^96^. However, the ionic conductivities at room temperatures (20–30 °C) are still two orders of magnitudes lower than that of conventional nonaqueous electrolytes, e.g., at 30 °C, ca. 2 × 10^−4^ (PolyIL-IS) vs. 1 × 10^−2^ S cm^−1^ (1.0 M LiPF_6_/ethylene carbonate-ethyl methyl carbonate^97^).

Although various approaches have been proposed, tested, and developed, true decoupled SPEs is still unattainable today. As C. Austen Angell mentioned in his 2019 review article, “there is some fundamental problem in the original salt-in-polymer solvent (and anionic polymer) physics, due to the ion proximity to the mobility-limiting polymer chains”^84^. This aspect is true for PEO-type SPEs and the anionic single-ion conductors (anionic transference number >0.9). This is also a fact in most PIS-type electrolytes since only a small portion of metal ions can be decoupled from the polymer, whereas the rest are still bound to the polymer chains. In the case of the PolyIL-IS systems, the weak coupling between the metal ion and the polymer exists through the anion-bridging co-coordination. The highly coupled metal ion-anion motion also limits the metal-ion transference number to ca. 0.5^95^. In this case, improving the ionicity of the salt could maximize the decoupling motion in PolyIL-IS, although not yet experimentally proved.

### SPE-based SSLMBs: from lab-scale development to practical applications and future directions

The development of SPE-based SSLMBs is closely associated with the discovery of positive electrode active materials. Lithium vanadium oxides (e.g., Li_1+*x*_V_3_O_8_, 0 <*x* < 2) have been long investigated as positive electrode active materials since the early 1970s due to their wide range of oxidation states enabling high specific capacities during reversible electrochemical reactions^98^. B. Scrosati and co-workers^99^ systematically studied the electrochemical performances of Li°|LiCF_3_SO_3_/PEO|Li_1.2_V_3_O_8_ cells and suggested that adequate cell rechargeability could be attained at relatively high temperatures (ca. 100 °C). With the optimization of SPEs and vanadium oxide-based positive electrodes, long-term (>1000 cycles) and stable cycling of these SPE-SSLMBs have been achieved by the research group at Hydro-Québec^100^. Yet, the main obstacle to large-scale implementation of batteries with vanadium-based positive electrodes lies, at the cell level, in the dissolution of vanadium species during continuous cycling, and at the raw material level, in the uneven geographical distribution of vanadium resources worldwide^101^.

**Fig. 7:**
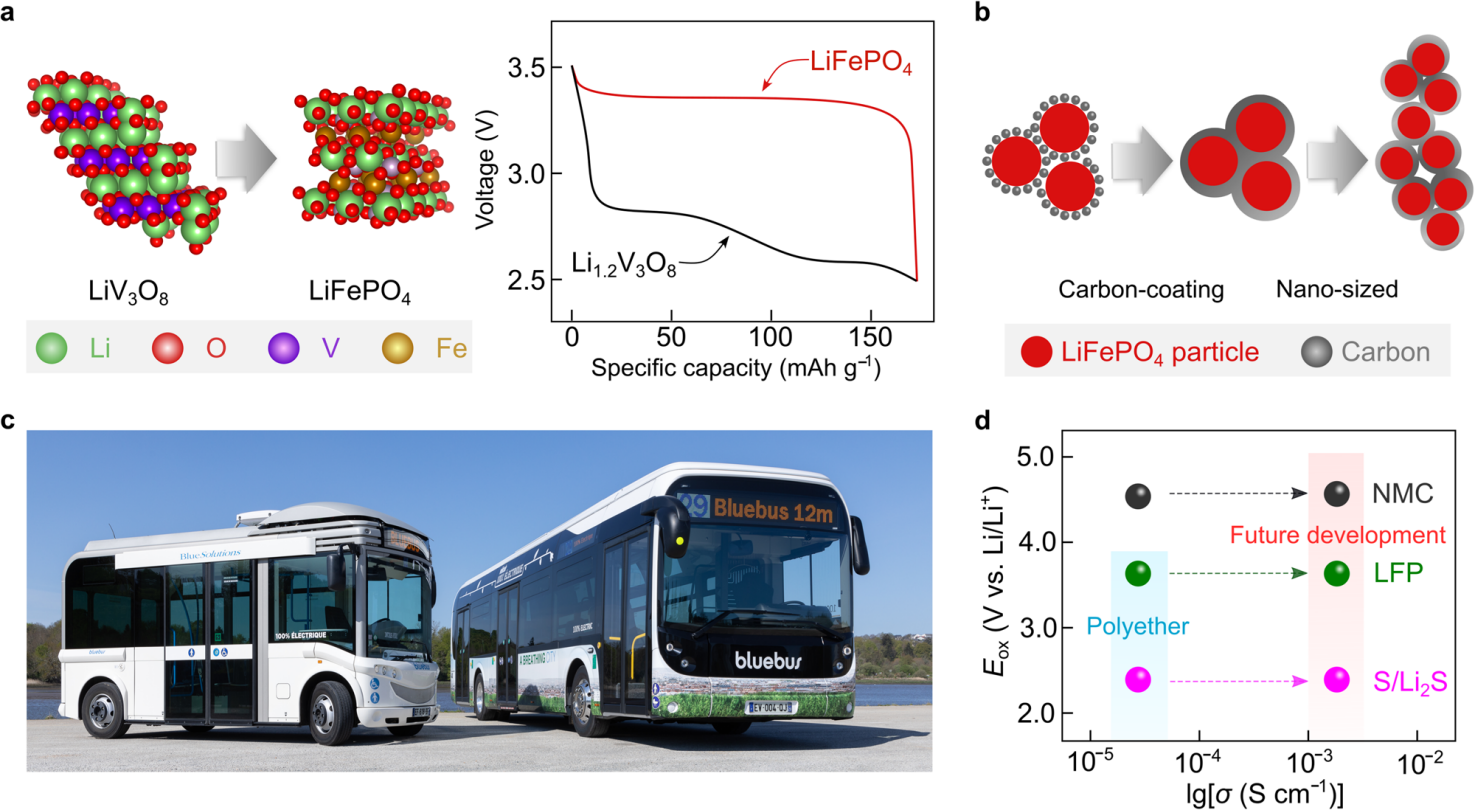
Implementation of positive electrode active materials and solid polymer electrolytes into practical applications. **a** (left) Schematic representation of the structural and chemical compositions of lithium vanadium oxide (LiV_3_O_8_) and lithium iron phosphate (LiFePO_4_, LFP). The crystal structures of LiV_3_O_8_ and LiFePO_4_ are obtained from Materials Projects^121^ and re-constructed with VESTA software^122^. (right) Comparison of voltage curves of Li metal cells with SPE comprising a Li_1.2_V_3_O_8_- and carbon-coated-LiFePO_4_- based positive electrodes cycled at 0.2 mA g^−1^. The values are taken from ref. ^101^. **b** Schematic representations of the LiFePO_4_ carbon-coating and nano-size reduction strategies **c** Photographic picture of the Bluebuses® developed by Bolloré group, in which SPE-based SSLMBs are utilized as the only power source. The photo is provided courtesy by the Bolloré Group. **d** Challenges for the contemporary SPEs and their batteries, in which conventional SPEs comprising polyethers are likely to be only suitable for low-voltage cathodes (e.g., sulfur (S), LFP), and improvement in the electrochemical window and ionic conductivities of SPEs are desired for future development.

In 1997, Padhi et al.^102^ reported an olivine-type positive electrode active material (Fig. 7a, left), i.e., lithium iron phosphate (LiFePO_4_), which shows a flat discharge/charge plateau at ca. 3.45 V vs. Li/Li^+^, and specific energy, at the material level, slightly higher than that of vanadium-based positive electrode active materials (Fig. 7a, right). However, the electronic conductivity of pristine LiFePO_4_ powder (e.g., 2 × 10^−9^ S cm^−1^ at 25 °C^103^) is lower compared to other layered oxide positive electrode active materials (e.g., 6 × 10^−4^ S cm^−1^ for lithium cobalt oxide (LiCoO_2_) at 25 °C^104^). This aspect favors sluggish electrode kinetics and poor rate-capability performance of the LiFePO_4_-based batteries. In 1998, Armand et al.^105^ attempted to increase the electrode kinetics by ball-milling LiFePO_4_ powder with conductive carbon in a poly(ethylene) jar. By accident, the ball-milling process was left for an unexpectedly long duration. The positive electrode formulated using the ball-milled LiFePO_4_ laminates showed improved enhanced rate-capabilities when tested in Li metal lab-scale cells using a nonaqueous electrolyte solution. Systematic investigations showed that ball-milled poly(ethylene) was converted into amorphous carbon during subsequent calcination and coated the surface of LiFePO_4_ particles. These results suggest that carbon-coating is an effective strategy to improve the reaction kinetics of positive electrode active materials with low electronic conductivity (Fig. 7b). Indeed, the deployment of carbon-coated LiFePO_4_ substantially increases the cycle life and attainable specific energy of SPE-based SSLMBs^105^. Moreover, the abundance of the elements in LiFePO_4_ suggests a higher sustainability of such technology than cobalt-based positive electrode active materials^14^. The rate performance of LiFePO_4_-based SSLMBs is further enhanced by decreasing the particle sizes of LiFePO_4_ powders into nanoscale prior to electrode formulation (Fig. 7b)^106,107^.

Presently, the SSLMBs comprising LiTFSI-based SPEs and LiFePO_4_-based positive electrodes have been practically deployed as power sources for EVs and grid storage by the Bolloré group. Figure 7c shows the photographic pictures of Bluebuses® equipped with 120 kWh SPE-based SSLMBs. Up to 2020, more than 1000 Bluecars® and 500 Bluebuses® have been produced, reaching a cumulated driving experience of >6 × 10^8^ km with a decent safety record (only two cases with unexplained runaway reactions). These industrially-relevant examples stimulated industrial and academic laboratories to restart the research activities in lithium metal rechargeable batteries after the initial abandonment of this technology as a consequence of the various fire accidents that occurred in AA-size Li°||molybdenum disulfide cells produced by Moly Energy in the late 1980s^24^.

It is important to highlight that a wider application of SPE-based SSLMBs is hindered by their low specific energy (<400 Wh kg^−1^) and rate-capability (< 2 C), stemming from the unsatisfactory anodic stability and ionic conductivities of the state-of-the-art SPEs (Fig. 7d). Besides, due to the low ionic conductivities of SPEs at room temperature (< 10^−4^ S cm^−1^ at 25 °C), the SPE-based SSLMBs need to be operated at elevated temperatures (60–80 °C), thus requiring additional accessories for thermal management^108^. To date, various types of solid electrolyte technologies have been proposed, e.g., structurally ordered organic/inorganic composite electrolytes^109^, in situ formed polymer electrolytes^110^, and localized high‐concentration electrolytes (i.e., with non-solvating diluents)^111^. Although these electrolyte concepts suggest plausible approaches to circumvent the obstacle to the development of solvent-free SPE-SSLMBs, further assessment of the long-term stability and scalability of these technologies are required to move toward technology readiness levels ≥5 (e.g., for practical application in large-format SSLMBs)^5^.

For the basic SPEs that simply contain a single lithium salt and a single polymer matrix, it is unclear if they could be used to boost the performances of SSLMBs. Although the anodic stability of the PEO-based SPEs is inadequate for coupling with high-voltage insertion-based positive electrode active materials (e.g., nickel-rich LiNi_*x*_Co_*y*_Mn_1−*x*−*y*_O_2_)^60^, it is possible to couple SPEs with high specific-energy positive electrode active materials that exploit conversion reactions for lithium-ion storage (e.g., sulfur/organosulfur and oxygen/lithium oxide). For example, Hu et al.^112^ proposed the utilization of iron fluoride (FeF_3_) as a conversion-type positive electrode to produce high-energy SPE-based SSLMBs. By introducing aluminum fluoride as an electrolyte additive, the *t*_Li_^+^ value of standard PEO-based SPEs was enhanced (ca. 0.6 at 60 °C), and the corresponding Li°||FeF_3_ lab-scale cell showed high initial capacities (ca. 600 mAh g^−1^ at 60 °C). In this context, it should also be pointed out that the multi-layer structure of electrolyte films could improve the stability of the interphases formed at the electrode|SPE interfaces. For instance, using a double-layer SPE comprising a polyether-based membrane for the lithium negative electrode and a polyester-based membrane for the high-voltage positive electrode could improve the cyclability of SSLMBs^113^. Moreover, the interdiffusion of lithium salt originating from the different activity coefficients (i.e., a measure for the difference between real and ideal solutions) in the two electrolytes in contact could be tailored by replacing discrete anions with polyanions^114^.

Looking at future development, to improve the rate-capability of SPE-based SSLMBs, it is essential to enchance the lithium-ion conductivities of SPEs by thinking out-of-the-box to deliver unconventional approaches for material development. For example, inheriting C. Austen Angell’s polymer-in-salt concept, would it be possible to prepare lithium salts with high ionicity in the liquid state at room temperatures (20–30 °C)? Starting from LiCF_3_SO_3_, the replacement of one oxygen atom ( = O) with =NSO_2_CF_3_ gives LiTFSI (i.e., Li[CF_3_SO_2_(NSO_2_CF_3_)]) with a lower melting point [i.e., *T*_m_ = 233 °C (LiTFSI) vs. >350 °C (LiCF_3_SO_3_)]^115^. A further homologation of the oxygen atom results in the formation of a super lithium sulfonimide salt (Li[CF_3_SO(NSO_2_CF_3_)_2_], LisTFSI) with a low melting transition approaching the ionic liquid domain (i.e., *T*_m_ ≤ 100 °C for typical ionic liquids^88^, and *T*_m_ = 118 °C for LisTFSI^116^). In this regard, we speculate that the concept of negative charge delocalization could be extended further to accessing liquid lithium salts. From another perspective, one may also replace typical neutral polyether/polyesters with charged polysalts (e.g., polycations, polyanions, or poly(zwitterions)), to regulate the ion-ion interactions and thereby achieving decoupled SPE systems^117^. For instance, the utilization of imidazole-type poly(zwitterions) could provide ordered subdomains with superionic nature, which allows rapid transport of ionic species even at temperatures close to their *T*_g_ values^118^.

In summary, we provided a reflection on SPEs and their application in SSLMBs focusing on the key milestones reached over the last five decades of research and development. The emergence of SPEs arises from the demand for soft solid electrolytes to circumvent the contact issues faced by SSLMBs using inorganic solid-state electrolytes. The utilization of SPEs stimulated the development of SSLMBs, making the long-term cycling of lithium metal negative electrodes possible. For future development, it is essential to bear in mind that designing high-performance SPE-based SSLMBs requires not only highly ionic conductive SPEs but also stable electrode|electrolyte interphases. As suggested by C. Austen Angell, decoupled SPE systems could be helpful in designing lithium salts and polymers with tailored structures and building lithium-ion conductive SPEs with high selectivity for cation transport. We advise that coupling high-energy conversion-type positive electrode active materials with SPEs could be an effective approach to enhance the energy content of the state-of-the-art SPE-based SSLMBs^5^, along with the improvement of inherent characteristics (e.g., suppressed volume changes, enhanced redox kinetics, high tap density, etc.) of conversion-type positive electrodes^5^. It has to be highlighted that SPEs with sufficient geometric flexibility are the key to confronting the solid-solid contact issues in SSLMBs^119^; and it could be anticipated that SPEs with improved electrochemical stabilities and ionic conductivities, particularly cation-only conductivities, will continue to be a desirable solution for developing more sustainable battery technologies.
